# Polyandry in the medfly - shifts in paternity mediated by sperm stratification and mixing

**DOI:** 10.1186/1471-2156-15-S2-S10

**Published:** 2014-12-01

**Authors:** Francesca Scolari, Boaz Yuval, Ludvik M Gomulski, Marc F Schetelig, Paolo Gabrieli, Federico Bassetti, Ernst A Wimmer, Anna R Malacrida, Giuliano Gasperi

**Affiliations:** 1 Department of Biology and Biotechnology, University of Pavia, Pavia, Italy; 2 Department of Entomology, The Hebrew University of Jerusalem, Rehovot 76100, Israel; 3 Justus-Liebig-University Gießen, Institute of Phytopathology and Applied Zoology, Heinrich-Buff-Ring 26-32, 35392 Giessen, Germany; 4 Department of Mathematics, University of Pavia, Pavia, Italy; 5 Department of Developmental Biology, Johann-Friedrich-Blumenbach Institute of Zoology and Anthropology, GZMB, Georg-August-Universität Göttingen, Ernst-Caspari-Haus, Justus-von-Liebig-Weg 11, 37077 Göttingen, Germany

**Keywords:** Medfly, polyandry, sperm stratification, transgenic sperm, fertilization chamber

## Abstract

**Background:**

In the Mediterranean fruit fly (medfly), *Ceratitis capitata*, a highly invasive agricultural pest species, polyandry, associated with sperm precedence, is a recurrent behaviour in the wild. The absence of tools for the unambiguous discrimination between competing sperm from different males in the complex female reproductive tract has strongly limited the understanding of mechanisms controlling sperm dynamics and use.

**Results:**

Here we use transgenic medfly lines expressing green or red fluorescent proteins in the spermatozoa, which can be easily observed and unambiguously differentiated within the female fertilization chamber. In twice-mated females, one day after the second mating, sperm from the first male appeared to be homogenously distributed all over the distal portion of each alveolus within the fertilization chamber, whereas sperm from the second male were clearly concentrated in the central portion of each alveolus. This distinct stratified sperm distribution was not maintained over time, as green and red sperm appeared homogeneously mixed seven days after the second mating. This dynamic sperm storage pattern is mirrored by the paternal contribution in the progeny of twice-mated females.

**Conclusions:**

Polyandrous medfly females, unlike *Drosophila*, conserve sperm from two different mates to fertilize their eggs. From an evolutionary point of view, the storage of sperm in a stratified pattern by medfly females may initially favour the fresher ejaculate from the second male. However, as the second male's sperm gradually becomes depleted, the sperm from the first male becomes increasingly available for fertilization. The accumulation of sperm from different males will increase the overall genetic variability of the offspring and will ultimately affect the effective population size. From an applicative point of view, the dynamics of sperm storage and their temporal use by a polyandrous female may have an impact on the Sterile Insect Technique (SIT). Indeed, even if the female's last mate is sterile, an increasing proportion of sperm from a previous mating with a fertile male may contribute to sire viable progeny.

## Background

The Mediterranean fruit fly (medfly), *Ceratitis capitata*, is a notorious pest with a worldwide distribution and a history of rapid expansion and devastating invasions [1,2]. The high reproductive capacity of this species is a key factor in its ability to rapidly colonize new areas. In the wild, polyandry associated with sperm precedence is a recurrent behaviour [3-6] that may represent an important adaptive trait. Remating by females sets the stage for post-copulatory sexual selection, which can shape both male and female biochemistry, physiology, morphology, and behaviour. As in many other species, a highly specialised female reproductive tract has evolved to deal efficiently with the sperm resources received from the males [7-11]. The medfly female's reproductive apparatus consists of a pair of ovaries, two short lateral oviducts joined by a short common oviduct to a long vagina, a pair of spermathecae and a pair of accessory glands. The anterior part of the vagina, that receives the sperm during copulation, contains the openings to the ducts that lead to the spermathecae and accessory glands. The spheroidal-shaped fertilization chamber lies ventrally in the anterior vagina and is characterized by the presence of about 70 small cavities called spermiophore alveoli, where the sperm are well placed to fertilize passing eggs [12,13]. The presence of multiple sperm storage organs potentially permits polyandrous females to manipulate ejaculates by segregating them into different locations and hence control the paternity of their progeny [14]. A number of studies have investigated the dynamics of sperm storage in the medfly in terms of relative amount of sperm stored in each organ and priority in fertilization. From a functional point of view, ablation experiments have established that the two spermathecae are long-term storage organs, whereas the fertilization chamber acts as staging point for sperm prior to their use in fertilization and is periodically replenished with sperm from the spermathecae [14].

Males have an intromittent organ (aedeagus) with three ejaculatory openings (gonopores). These three gonopores are thought to permit sperm emission towards the two spermathecae and the fertilization chamber, suggesting that sperm are injected into the three female sperm storage compartments simultaneously [13,15]. Both types of sperm storage organs are able to maintain sperm viability, as more than 80% of the stored sperm transferred to females can survive in both organs for at least 18 days following insemination [14]. In addition, several studies have focused on the pattern of sperm precedence, in terms of the proportion of progeny sired by the second of two males (P2) following female remating [3-6,16]. Recently, we correlated estimates from sperm counts in the female storage organs with paternity in twice-mated females [15]. We found a significant advantage of the second male's sperm in siring progeny [15]. Strikingly however, we found that patterns of paternity in multiply mated females are dynamic - second male sperm precedence (P2) decreases in favour of the first male (P1) as a function of time. This temporal increase in the proportion of offspring sired by the first male may be the result of the way the sperm of successive males are stored in the female reproductive tract. We thus proposed a model for which sperm from the first and the second male are stored in a somehow stratified fashion, without immediate mixing of the two ejaculates, so that those that enter later are used earlier in fertilization [15].

To date, understanding the mechanisms involved in medfly sperm use has been constrained by the technical challenges of directly observing sperm dynamics within the female reproductive tract and our limited ability to discriminate between sperm of different males.

Thus, here we use transgenic medfly lines expressing green or red fluorescent proteins in the spermatozoa [17], which can be easily observed and unambiguously differentiated within the female fertilization chamber. We tested and validated the hypothesis that it is the order and time-line of sperm storage to determine its subsequent use by twice-mated females.

## Methods

The experimental strategy involves: (1) the choice of appropriate transgenic medfly lines. (2) The set-up of mating and remating tests. (3) The assessment of paternity in females mated successively to two males. (4) Statistical analyses. (5) Confocal and stereomicroscopic visualization of dissected fertilization chambers from twice-mated females to directly observe transgenic sperm distribution at different times following insemination.

### Medfly lines

Three different medfly lines were used: i) the long-established laboratory strain Egypt-II (EgII), obtained from the FAO/IAEA Insect Pest Control Laboratory (Seibersdorf, Austria) and two molecularly and functionally characterized and critically evaluated homozygous transgenic strains generated by *piggyBac*-mediated germline transformation, namely ii) #1260_F-3_m-1 (tGFP1) and iii) #1261_F-5_m-5 (DsRedEx1) [17]. Both transgenic strains carry a body- and a sperm-specific marking system. The sperm-specific marker systems were generated by fusing the medfly *β2-tubulin *promoter (*Ccβ2t*) to turboGFP (*tGFP*) or DsRedExpress (*DsRedEx*) genes which were then used to engineer constructs based on *piggyBac *vectors carrying polyubiquitin (PUb)-driven EGFP or DsRed germline transformation markers, respectively. Specifically, males from the tGFP1 line express *Ccβ2t *promoter-driven tGFP in the testes/sperm and the Pub-DsRed marker produces red fluorescence in the body. Conversely, males from the DsRedEx1 line express *Ccβ2t *promoter-driven DsRedEx in the testes/sperm and green fluorescence in the body because of the presence of the Pub-EGFP marker. In both transgenic lines, transgene insertions are single transposon integrations as proven by Southern hybridisation and inverse PCR [17] and fluorescence of all markers has remained stable for more than 90 generations in our insectary. The two transgenic lines display very similar hatching rates [17].

Wild-type and transgenic medfly lines were maintained under standard rearing conditions [18].

### Mating experiments

EgII, tGFP1, and DsRedEx1 adult flies of similar size were sexed by chilling at emergence and maintained under the same nutritional and environmental conditions. Mating and remating tests were performed in the ratio 1:3 (1 wild-type female : 3 transgenic males). In total, about 100 females and 300 males for first mating assays and 70 females plus 200 males for remating assays were used. Virgin females and males (48 h old) were used for the first mating tests. To assess any effects attributable to differential sperm storage and use, some wild-type EgII females were mated to tGFP1 and then to DsRedEx1 males, and others first to DsRedEx1 and then to tGFP1 males. The remating tests were performed 24 hours after the first mating, by exposing the females to virgin males of the same age [15]. For both mating and remating tests, pair formation was monitored for four hours and copulating pairs were coaxed into a test tube. Only females that remained in *copula *for at least 100 min were used in the following assays [19]. Single-mated females were dissected to assess the fluorescent sperm distribution within their fertilization chamber. For twice-mated females, flies were either dissected to observe sperm distribution in the fertilization chamber or transferred immediately after the remating to single cages to allow for oviposition. Daily ovipositions from these females (24 h collections) were collected separately and reared to adults for paternity assignment.

### Paternity assay

The proportion of progeny sired by the first (P1) and second (P2) male was determined on the offspring of 102 twice-mated EgII females, 52 of which were mated first to tGFP1 and then to DsRedEx1 males (overall progeny n = 7530) and 50 mated first to DsRedEx1 and then to tGFP1 males (overall progeny n = 4882). For each twice-mated female, seven daily ovipositions were analysed to monitor the paternity trend over time. This time frame corresponds to the medfly steady state period of constant rate of egg-laying [20]. We considered only ovipositions that resulted in at least ten adult progeny. For each oviposition day, the total progeny sired by each female were screened by epifluorescence for the expression of *PUb-DsRed, PUb-EGFP, Ccβ2t-tGFP *and *Ccβ2t-DsRedEx *using an Olympus SZX7 fluorescence stereomicroscope with the filter set SZX-MGFP for the detection of green (cod. 33775; EX 460-490; DM 505; BA 510IF) and SZX-MIY for red fluorescence (cod. 33778; EX 540-580; DM 600; BA 610IF). The proportion of progeny that were sired by the first (P1) or second male (P2) was then calculated.

### Statistical analysis

We analysed the paternity data using a logistic regression (generalized linear model with binomial errors) in the two reciprocal mating/remating crosses separately. We thus fitted the following model for *p *as a function of *x*:

$$p\left(x\right)=\frac{e^{\left(a+bx\right)}}{1+e^{\left(a+bx\right)}}$$

*p *represents the proportion of progeny attributable to the first male (P1), *x *corresponds to the oviposition day, *a *and *b *to the intercept and the slope of the graph of P1 against oviposition day, respectively. Statistical analyses were performed using R project software, v2.2.1 (http://www.r-project.org/).

### Light and fluorescence microscopy

The fertilization chambers of once- and twice-mated females were dissected in PBS one day and seven days after the last copula. Fresh samples were mounted on glass slides in PBS and analysed with an Axioplan (Zeiss) epifluorescent microscope. Filter sets used for the screening of tGFP and DsRedEx were the Zeiss filter set 13 (Ex. 470/20; Em. 505-530) and filter set 20 (Ex. 546/12; Em. 575-640), respectively. Images were captured with a 40x objective using an Olympus DP70 digital camera.

### Confocal microscopy

Fluorescent confocal microscopy was used to examine and to resolve the details of the fertilization chambers dissected from once- and twice-mated females. Images were obtained from a Leica TCS-SP5 II system mounted on a Leica DMIRBE inverted microscope. Spaced optical sections were recorded using a 63x oil immersion objective. Images were collected in the 1024 × 1024 pixel format and processed by the Leica Confocal Software. Green and red fluorescent images were captured simultaneously and merged into a new image to visualize the localisation of red and green sperm.

## Results

### Sperm of the first male are under-represented in the initial progeny of twice-mated females, independently of the transgenic male order

The overall number of progeny produced by the 52 females mated first to the tGFP1 and then to the DsRedEx1 males (n = 7530), was significantly greater than those produced by the 50 females in the reciprocal mating cross (n = 4882)(Wilcoxon rank sum test with continuity correction *P *= 3.06e-6). Moreover, in the females first mated to DsRedEx1 and then to tGFP1 males, the mean number of progeny per female, in the first two oviposition days, was significantly lower compared to that in the following five oviposition days (Wilcoxon rank sum test with continuity correction, *P *= 8.76e-10). However, in the reciprocal cross there was no significant difference in these two oviposition intervals (Wilcoxon rank sum test with continuity correction, *P *= 0.07).

In both remating tests, the proportion of progeny sired by the first male (P1) is under-represented in the cumulative progeny of the first seven days. Indeed, the combined data from the 52 females mated first to tGFP1 and then to DsRedEx1 males indicated that, during the first seven days following the second copulation, the first male (tGFP1) sired 33% (P1) and the second male (DsRedEx1) sired 67% (P2) of the total progeny. In the reciprocal cross, P1 (attributable to DsRedEx1) was 31%, while P2 (tGFP1) was 69% of the total progeny (Table 1).

**Table 1:** Proportion of progeny sired by the first male (P1) in relation to oviposition day.

	Oviposition day							
Male parental combination	Day 1	Day 2	Day 3	Day 4	Day 5	Day 6	Day 7	Overall
**tGFP1, DsRedEx1**								
No. of ovipositing females	50	34	50	39	43	50	40	
No. of progeny	1192	782	1198	1151	1099	1145	963	7530
Mean progeny/female ± SE	23.8 ± 1.83	23.0 ± 1.85	24.0 ± 1.32	29.5 ± 2.37	25.6 ± 1.53	22.9 ± 1.06	24.1 ± 1.56	
Mean P1 ± SE	0.31 ± 0.03	0.29 ± 0.04	0.32 ± 0.04	0.30 ± 0.05	0.33 ± 0.05	0.36 ± 0.05	0.42 ± 0.06	0.33 ± 0.02
**DsRedEx1,tGFP1**								
No. of ovipositing females	41	23	27	40	36	36	36	
No. of progeny	611	367	482	1077	746	875	724	4882
Mean progeny/female ± SE	14.9 ± 0.46	15.9 ± 1.10	17.9 ± 1.44	26.9 ± 1.95	20.7 ± 1.15	24.3 ± 1.21	20.1 ± 1.45	
Mean P1 ± SE	0.16 ± 0.03	0.13 ± 0.02	0.32 ± 0.05	0.35 ± 0.05	0.31 ± 0.05	0.46 ± 0.06	0.43 ± 0.06	0.31 ± 0.05

### First male sperm use increases over time

**Figure 1:**
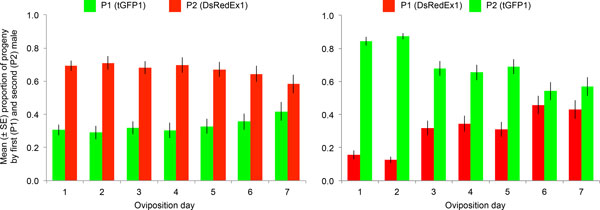
**Changes in the proportion of offspring sired by the first (P1) and second (P2) male over time from twice-mated females**. The mean proportion of progeny sired by 52 wild-type females mated first to tGFP1 males and then to DsRedEx1 males is shown on the left, whereas the mean proportion of progeny sired by 50 females mated according to the reciprocal male order is shown on the right. Green bars represent the progeny attributable to tGFP1, whereas red bars represent the progeny attributable to DsRedEx1 males. Vertical bars indicate standard errors.

Twice-mated females were also used to obtain a distinct record of paternity for each of the first seven oviposition days (Table 1 and Figure 1). In both reciprocal crosses, the average first male paternity (P1) showed a tendency to increase as more eggs were laid, from 31% on the first day, up to 42% on the seventh oviposition day when tGFP1 males were the first mate, and from 16% to 43% when DsRedEx1 males acted as first mate. To determine whether this apparent trend is statistically significant, a logistic regression analysis was performed using a generalized linear model with binomial errors. The overall regression model slope, corresponding to the oviposition day, was significantly different from zero when the DsRedEx1 males acted as first mate (*z *= 11.87, *P *< 2e-16), whereas marginally non-significant when the tGFP1 males acted as first mate (*z *= 1.798, *P *= 0.0722). Indeed, in the DsRedEx1,tGFP1 remating parental combination, the P1 values in the first two oviposition days were significantly lower than in the following five days (Wilcoxon rank sum test with continuity correction, *P *= 4.9e-7), whereas no significant difference was detected in the reciprocal cross (*P *= 0.80).

### Direct visualization of sperm dynamics in the fertilization chamber of twice-mated females

**Figure 2:**
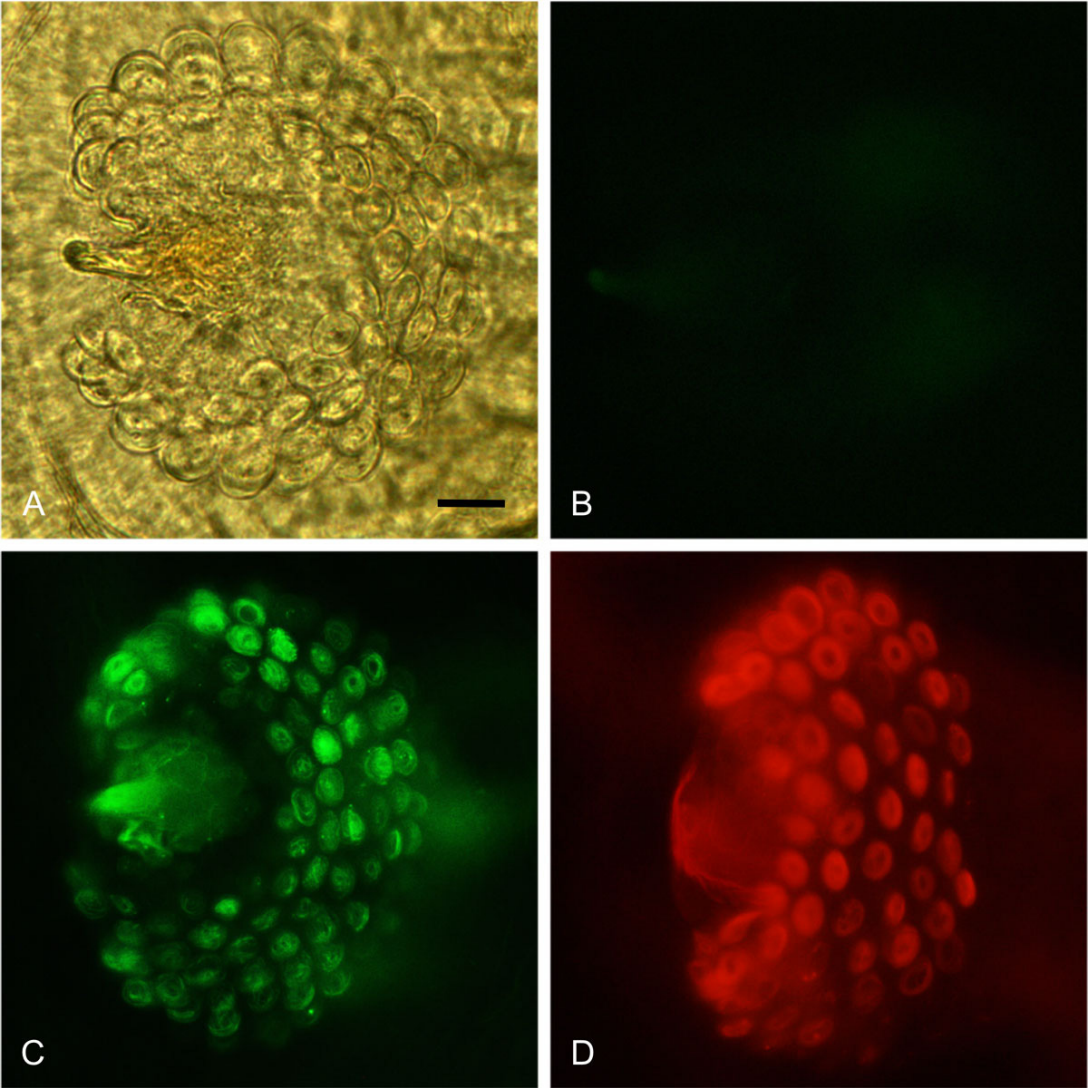
**Fertilization chambers of once-mated females**. The fertilization chambers were dissected from wild-type females once-mated to wild-type (2A-B), tGFP1 (2C), and DsRedEx1 (2D) males, respectively. Picture 2A was captured using phase contrast, whereas Pictures 2B, 2C and 2D are the result of merging of phase contrast and epifluorescence microscopy captured with the Zeiss filters sets 13 and 20. Scale bar = 15 µm.

**Figure 3:**
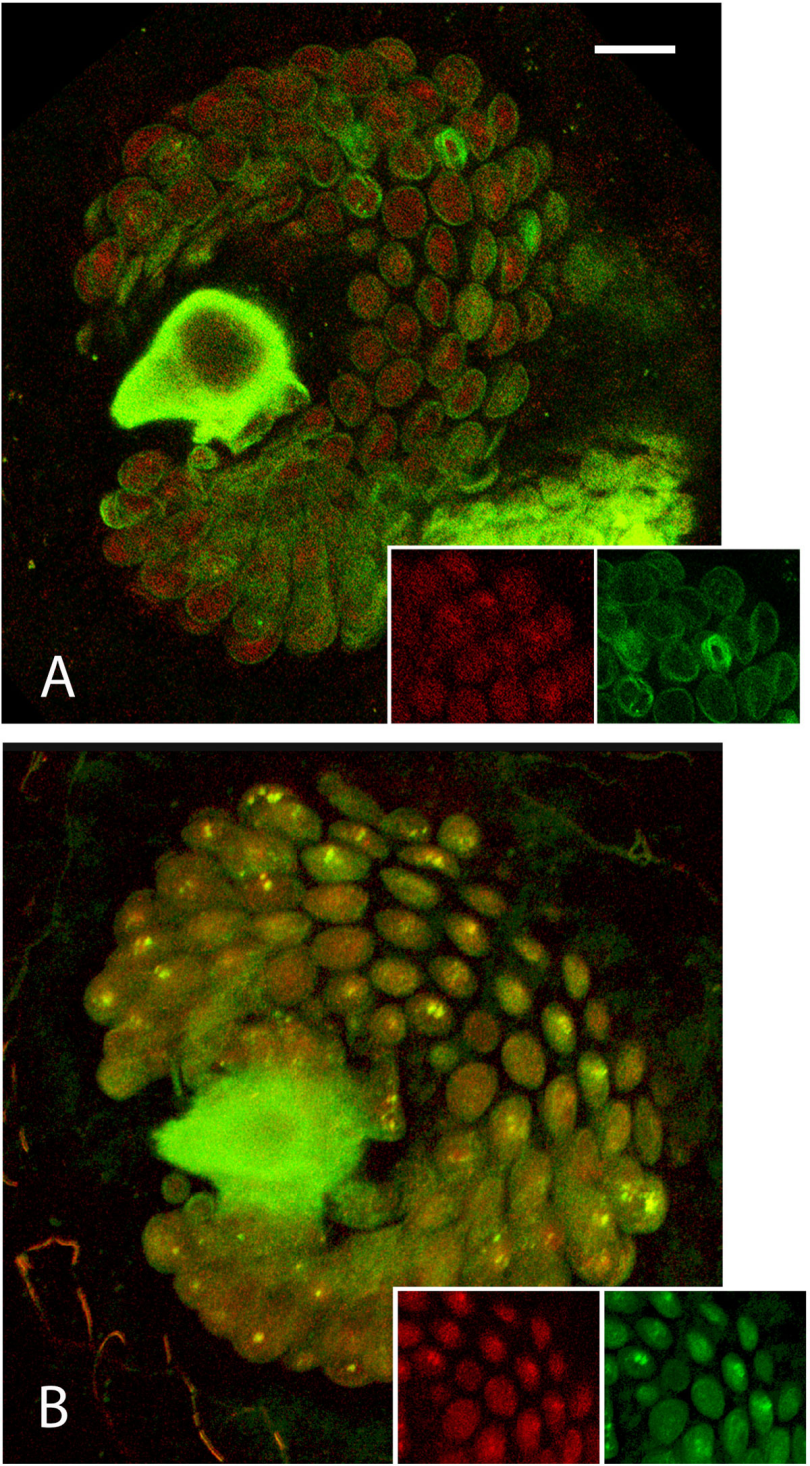
**Confocal merged images of fertilization chambers of twice-mated females**. Green sperm was transferred by the first male (tGFP1 line) and the red sperm (DsRedEx1 line) by the second male. In 3A, the fertilization chamber was dissected 24h after the remating and immediately observed. In 3B, the fertilization chamber was dissected seven days after the remating. The two squares at the bottom of each picture show, from the left, the single red and green unmerged images, respectively. Scale bar = 15 µm.

We used both an epifluorescence and a confocal microscope-based approach to visualize the dynamics of the transgenic green and red sperm within the fertilization chamber of either once- and twice-mated wild-type females. In the fertilization chamber of once-mated females, either tGFP1 or DsRedEx1 sperm appeared to be clearly distributed in all the alveoli, where numerous bundles of coiled spermatozoa were visible (Figure 2). In the fertilization chambers of twice-mated females 24 h after the second copulation, the sperm from the first male appeared to be homogenously distributed all over the distal portion of each alveolus, whereas sperm from the second male were clearly concentrated in the central portion of each alveolus (Figure 3A). This distribution was evident in both the reciprocal parental male combinations. Seven days after remating, this distinct stratified sperm distribution was no longer evident. The green and the red sperm were homogeneously mixed, occupying the complete cavity of each alveolus (Figure 3B).

## Discussion

Here we provide clear support that polyandrous medfly females, unlike *Drosophila *[21-23], conserve sperm from two different mates to fertilize their eggs. The female initially stores the sperm from the first and second male in the fertilization chamber in a stratified fashion, with subsequent sperm mixing. The presence of evident sperm stratification in the fertilization chamber one day after remating explains the initially low P1 values. These results exclude a role of sperm displacement or dumping mechanisms that are present in *Drosophila *[21-23], yet seem be due to strategic insemination by males and the sperm dynamics within the female storage organs. When the egg enters the anterior vagina with its micropyle towards the fertilization chamber entrance, the sperm from the chamber are the first to be used for fertilization and, given the clear stratification that we documented (Figure 3), the last sperm to enter the chamber are the first to be used for fertilization, accounting for a P2 value of roughly 70% of the total progeny. The gradual increase in P1 in the following days may be explained by the progressive exhaustion of the sperm in the fertilization chamber and the contemporary arrival of other sperm from the spermathecae, re-filling the spermiophore alveoli [15]. The microscope images are consistent with our paternity results, since we show that in the first days after remating P1 increases, suggesting the arrival of spermathecal sperm to replenish the chamber, resulting in an increase in sperm mixing. In addition, these results are consistent with the sperm dynamics recorded by Twig and Yuval [14]. They noted that although there is a significant drop in sperm numbers in the fertilization chamber between one and three days after mating, subsequently the level of sperm in the chamber remains quite constant, suggesting replenishment from the spermathecae. As further support, in other tephritids similar patterns have been observed [24-26].

In terms of fertilization success, the sperm of two transgenic lines used in this study displayed a similar efficiency in siring their overall P1 and P2 offspring (P1 = 33 and 31%; P2 = 67 and 69%, in the two reciprocal crosses, respectively). This data, together with the results from a previous study [17], suggest the absence of major dysfunctions of the transformed sperm.

However, in the remating tests we observed that, when tGFP1 was the first mate, the increase in P1 was less evident than in the reciprocal cross. This may be due to differences in the amount of sperm transferred by tGFP1 with respect to DsRedEx1 males (1854 ± 232 (SE) and 1164 ± 176 (SE), respectively), as previously assessed [17]. We suggest that this difference in sperm transfer rates may have a stronger effect in the first days after the remating. The more abundant tGFP1 first male sperm may indeed reflect the high P1 values, which are twice the P1 attributable to DsRedEx1 males in the reciprocal cross, both at day 1 and day 2 after remating.

## Conclusions

Our findings represent an advance in understanding the complex reproductive biology of this highly invasive pest [2,27]. We confirm that the sperm load from a single mating does not exploit the storage capacity of the female sperm storage organs, mirroring the strategic partitioning of the male's sperm reserves among different females to optimize his reproductive success [28-31]. As a reproductive strategy, the sperm stratification we observed may initially favour the fresher ejaculate from the second male. However, as his sperm gradually becomes depleted, the sperm from the first male becomes increasingly available for fertilization. Despite the relatively high longevity of medfly sperm [14,17], the freshest sperm will most probably have a higher viability compared to that from the first male partner. The utilisation of sperm from different male partners in the medfly represents an adaptive strategy to maintain genetic variability in populations arising from new invasions or bottlenecks [2,15]. Given that medfly adults disperse over large distances in search of suitable hosts, young females need to fill their sperm reserves [3,5,6,32,33] to ensure egg fertilization long after dispersal. As a consequence, colonizing polyandrous females can benefit their descendants through a reduction in the cost of inbreeding since matings among their progeny will tend to be between half siblings as well as between full siblings. This favors a rapid increase in the effective population size and provides selective advantages in limiting the erosion of genetic diversity [15].

As in other insect species, male competition for fertilization success may be a multivariate process involving ejaculate-female and ejaculate-ejaculate interactions, as well as complex sperm behaviour *in vivo *[21,34,35]. Studies on the effects of male accessory secretions on female physiology and fertilization dynamics have been initiated [36], and may provide the key to understanding the subtle differences between strains in paternity, and how sperm mobilization from the spermathecae to fertilization chamber is regulated.

Knowledge on the dynamics of sperm storage and use will have a major impact on the application of Sterile Insect Technique (SIT) for pest control [37]. It is widely known that sterile males display lower mating competitiveness than wild males [38,39]. In addition, it has been repeatedly observed that females mated first to sterile males display higher remating frequencies than those mated to normal males [6,40-44]. According to our findings on the mechanisms of sperm storage and use, approaches based on the SIT could be less efficient in the presence of polyandry, since, even if the last male to mate a female is sterile, an increasing proportion of sperm from a previous mating with a fertile male may contribute to sire viable progeny. For this reason, further experiments to clarify the outcome of matings between wild-type females and sterile/fertile males as first/second mates are needed. In any case, the efficiency of the SIT can be improved by increasing the ratio of sterile males to wild fertile males, although accurate modelling will be needed to determine the optimal balance between the wild population and the sterile males released. As a last consideration, the use of sterile strains with easily recognizable, labelled, sperm will be invaluable for monitoring their competitiveness and hence the success of the release programme.

## Competing interests

The authors declare that they have no competing interests.

## Authors' contributions

Conceived and designed the experiments: FS, BY, MFS, EAW, ARM, GG. Performed the experiments: FS, PG. Analysed the data: FS, LMG, MFS, FB. Wrote the paper: FS, LMG, BY, MFS, ARM, GG. All authors read and approved the final manuscript.
